# The soluble guanylate cyclase activator cinaciguat prevents cardiac dysfunction in a rat model of type-1 diabetes mellitus

**DOI:** 10.1186/s12933-015-0309-x

**Published:** 2015-10-31

**Authors:** Csaba Mátyás, Balázs Tamás Németh, Attila Oláh, László Hidi, Ede Birtalan, Dalma Kellermayer, Mihály Ruppert, Sevil Korkmaz-Icöz, Gábor Kökény, Eszter Mária Horváth, Gábor Szabó, Béla Merkely, Tamás Radovits

**Affiliations:** Heart and Vascular Center, Semmelweis University, Városmajor u. 68., Budapest, 1122 Hungary; Experimental Laboratory of Cardiac Surgery, Department of Cardiac Surgery, University of Heidelberg, INF 326. OG 2, 69120 Heidelberg, Germany; Institute of Pathophysiology, Semmelweis University, Nagyvárad tér 4., Budapest, 1089 Hungary; Institute of Human Physiology and Clinical Experimental Research, Semmelweis University, Tűzoltó u. 37-47., Budapest, 1094 Hungary

**Keywords:** Heart failure, cGMP, Pressure–volume relationship, sGC activator, Diabetic complications, Insulin dependent diabetes mellitus, Oxidative stress, PDE-5, Apoptosis, Fibrosis

## Abstract

**Background:**

Diabetes mellitus (DM) leads to the development of diabetic cardiomyopathy, which is associated with altered nitric oxide (NO)—soluble guanylate cyclase (sGC)—cyclic guanosine monophosphate (cGMP) signalling. Cardioprotective effects of elevated intracellular cGMP-levels have been described in different heart diseases. In the current study we aimed at investigating the effects of pharmacological activation of sGC in diabetic cardiomyopathy.

**Methods:**

Type-1 DM was induced in rats by streptozotocin. Animals were treated either with the sGC activator cinaciguat (10 mg/kg/day) or with placebo orally for 8 weeks. Left ventricular (LV) pressure–volume (P–V) analysis was used to assess cardiac performance. Additionally, gene expression (qRT-PCR) and protein expression analysis (western blot) were performed. Cardiac structure, markers of fibrotic remodelling and DNA damage were examined by histology, immunohistochemistry and TUNEL assay, respectively.

**Results:**

DM was associated with deteriorated cGMP signalling in the myocardium (elevated phosphodiesterase-5 expression, lower cGMP-level and impaired PKG activity). Cardiomyocyte hypertrophy, fibrotic remodelling and DNA fragmentation were present in DM that was associated with impaired LV contractility (preload recruitable stroke work (PRSW): 49.5 ± 3.3 vs. 83.0 ± 5.5 mmHg, P < 0.05) and diastolic function (time constant of LV pressure decay (Tau): 17.3 ± 0.8 vs. 10.3 ± 0.3 ms, P < 0.05). Cinaciguat treatment effectively prevented DM related molecular, histological alterations and significantly improved systolic (PRSW: 66.8 ± 3.6 mmHg) and diastolic (Tau: 14.9 ± 0.6 ms) function.

**Conclusions:**

Cinaciguat prevented structural, molecular alterations and improved cardiac performance of the diabetic heart. Pharmacological activation of sGC might represent a new therapy approach for diabetic cardiomyopathy.

**Electronic supplementary material:**

The online version of this article (doi:10.1186/s12933-015-0309-x) contains supplementary material, which is available to authorized users.

## Background

Diabetes mellitus (DM) is associated with cardiovascular complications, such as myocardial infarction, chronic heart failure or vascular diseases. It acts as an independent risk factor for coronary atherosclerosis and ischemic heart disease, however, the altered metabolic state—due to elevated glucose levels—has a direct impact on cardiac structure and function independently of coronary artery disease. Thus DM leads to the development of a special disease entity, termed diabetic cardiomyopathy [1]. Although, both systolic and diastolic dysfunction [2–5] as well as several key morphological and cellular/subcellular features (including myocardial fibrotic remodelling, cardiomyocyte hypertrophy, nitro-oxidative stress, inflammation and apoptosis) have been described in diabetic cardiomyopathy, the exact mechanisms in the pathophysiology are still unknown [1, 6–9].

In the cardiovascular system, under physiological conditions, nitric oxide (NO) is produced by the endothelial nitric oxide synthase (eNOS) in endothelial cells and diffuses into target cells, such as vascular smooth muscle cells or cardiomyocytes. It activates its intracellular receptor, the soluble guanylate cyclase enzyme (sGC) which results in the rapid formation of the second messenger cyclic guanosine monophosphate (cGMP). cGMP activates the cGMP-dependent protein kinase (PKG), which mediates most of its physiological effects, such as vasodilatation or inhibition of platelet aggregation [10]. The NO–sGC–cGMP–PKG signalling pathway has been described to get disturbed in DM through several mechanisms, such as increased formation of reactive nitrogen (RNS) and oxygen species (ROS) (nitro-oxidative stress), eNOS uncoupling and decreased NO bioavailability, increased expression of the cGMP-degrading enzyme phosphodiesterase (PDE)-5 [1]. Our research group and others demonstrated protective effects of enhanced cGMP-signalling via pharmacological inhibition of PDE-5 in several cardiovascular diseases [11–15] and in DM in particular [3, 16].

Nitro-oxidative stress directly deteriorates the structure of sGC resulting in a heme-deficient, NO-insensitive and inactive form of the enzyme [17]. Therefore, drugs that are able to activate the NO-insensitive form of sGC thus reactivating it might have cardioprotective effects in various pathological conditions through the enhancement of the impaired cGMP-signalling. The sGC activator cinaciguat (BAY 58-2667) has been reported to bind to the heme pocket of NO-insensitive sGC [17] thus increasing its cGMP-producing activity and augmenting cGMP-PKG signalling. Recent studies described its beneficial effects in experimental myocardial infarction [18], myocardial ischemia/reperfusion injury [19, 20], endothelial dysfunction induced by nitro-oxidative stress [21], vascular neointima formation [22] or in prevention of cardiomyocyte hypertrophy [23].

The safety and tolerability of cinaciguat have been assessed by phase-I human clinical trials [24]. Although cinaciguat has demonstrated efficacy in a proof-of-concept study in patients with acute decompensated heart failure [25], the phase-IIb clinical studies for the same indication has been terminated prematurely due to the high number of adverse events (mainly a significant drop of blood pressure) during acute intravenous application [26, 27]. The investigators concluded that rather a chronic, long term oral administration of the compound could improve endothelial and preserve myocardial function in heart failure patients [28].

Based upon the concept of cardioprotection by enhanced cGMP-signalling via chronic pharmacological activation of the sGC enzyme, we investigated in the present study whether long-term administration of the sGC-activator cinaciguat can preserve left ventricular (LV) systolic and diastolic function and prevent DM-associated myocardial alterations in the rat model of streptozotocin (STZ)-induced experimental type-1 DM.

## Methods

### Animals

The investigation conformed to the EU Directive 2010/63/EU guidelines and the Guide for the Care and Use of Laboratory Animals published by the US National Institutes of Health (NIH Publication No. 85–23, revised 1996) and was reviewed and approved by the appropriate institutional and national ethical committees (permission number: 22.1/1162/3/2010). All animals received humane care.

8-week-old male Sprague–Dawley rats (n = 46; body weight (BW): 225–250 g) (Charles River, Sulzfeld, Germany) were housed individually in a room (constant temperature, 12/12 h light/dark cycles) and received standard laboratory rat diet and water ad libitum.

### Induction of DM

Type-1 DM was induced with a single i.p. injection of STZ (60 mg/kg) in our rats as described previously [3]. STZ was freshly dissolved in citrate buffer (0.1 M). Control animals received only the buffer. 72 h after the injection a drop of blood was collected from the tail vein and blood glucose concentration was determined by using a digital blood glucose meter (Accu-Chek^®^ Sensor, Roche, Mannheim, Germany). Animals with a random blood glucose level >15 mmol/l were considered to be diabetic and were included into the study.

### Experimental groups, chronic treatment protocol

After confirmation of DM, rats were randomised into four groups: vehicle-treated control (Co; n = 12), cinaciguat-treated control (CoCin; n = 12), vehicle-treated diabetic (DiabCo; n = 12) and cinaciguat-treated diabetic (DiabCin; n = 10) groups. Animals were treated for 8 weeks with 0.5 % methylcellulose (Co, DiabCo) vehicle or with the sGC activator cinaciguat in suspension p.o. (CoCin, DiabCin; 10 mg/kg/day), starting immeadiately after DM confirmation. Water bottles were filled every morning with the same amount of fresh tap water and daily water intake was measured. Animal cages were handled with care and were not moved after water bottle replacement to prevent spilling of water from the bottles. Body weight of the animals were recorded every 2 days and the dose of cinaciguat was adjusted accordingly.

### Hemodynamic measurements

24–28 h after the last administration of cinaciguat/vehicle, LV pressure–volume (P–V) analyses were carried out using a pressure-conductance microcatheter system (MPVS-Ultra, Millar Instruments, Houston, TX, USA) to evaluate LV performance. Animals were anesthetised with a mixture of xylazine (3 mg/kg) and ketamin (100 mg/kg) i.p. [3, 29]. Mean arterial blood pressure (MAP), heart rate (HR), maximal LV systolic pressure (LVSP), LV end-diastolic pressure (LVEDP), maximal slope of systolic pressure increment (dP/dt_max_) and diastolic pressure decrement (dP/dt_min_), time constant of LV pressure decay (Tau), ejection fraction (EF), stroke work (SW) and cardiac output (CO) were calculated. The slope (E_es_) of the LV end-systolic P–V relationships (ESPVR; according to the parabolic curvilinear model) and preload recruitable stroke work (PRSW) were calculated as load-independent indexes of LV contractility while the slope of the LV end-diastolic P–V relationship (EDPVR) was calculated as reliable index of LV diastolic stiffness. For detailed hemodynamic description see Additional file 1.

### Biochemical measurements

A drop of blood was collected from the tail vein and blood glucose concentration was determined using a digital blood glucose meter (Accu-Chek^®^ Sensor, Roche). At the end of the hemodynamic measurements blood was collected from the inferior caval vein, plasma samples were prepared and stored at −80 °C.

Plasma cGMP levels were determined by enzyme immunoassay (EIA) using a commercial kit (Amersham cGMP EIA Biotrak System, GE Healthcare, Buckinghamshire, UK).

### Myocardial mRNA analysis

We performed quantitative real-time polymerase chain reaction (qRT-PCR) experiments as described previously [30]. Frozen LV samples were homogenised, total RNA was isolated. Reverse transcription was performed and cDNA samples were amplified on the StepOnePlus™ Real-Time PCR System (Applied Biosystems, Foster City, CA, USA) using TaqMan^®^ Universal PCR MasterMix and TaqMan^®^ Gene Expression Assays (Applied Biosystems) for the following targets: atrial natriuretic factor (ANF), α myosin heavy chain (MHC) and β-MHC (ratio of β/α-MHC expression was assessed as pathological cardiomyocyte hypertrophy marker), antiapoptotic mediator B cell CLL/lymphoma-2 (Bcl-2), proaptotic mediator Bcl2-associated X protein (BAX), eNOS, mediators of cardiac remodelling such as collagen 1a1 (Col1), 3a1 (Col3) and fibronectin, matrix metallopeptidase (MMP)-2 and MMP-9 and their endogenous inhibitors tissue inhibitor of MMP (TIMP)-1 and TIMP-2, members of different antioxidant systems like catalase, thioredoxin-1, gluthatione-reductase, superoxide dismutase (SOD)-2 and heat shock 70 kD protein 1A (HSP70a1). Gene expression data were normalised to glyceraldehyde-3-phosphate dehydrogenase (GAPDH). The mRNA expression levels were calculated using the CT comparative method (2^−ΔCT^) and adjusted to a positive calibrator. For detailed description see Additional file 1.

### Immunoblot analysis

LV tissue samples were homogenised, protein concentration was determined and equal amounts of protein were separated via gel-electrophoresis. Proteins were transferred to nitrocellulose membranes. After blocking, membranes were incubated with primary antibodies against various targets: eNOS (1:1000, SC-654, SantaCruz Biotechnology, Santa Cruz, CA, USA), sGC β1 (1:1000, NB100-91798, Novus Biologicals, Cambridge, UK), PDE-5 (1:2000, ALX-210-099, Enzo Life Sciences, Farmingdale, NY, USA), PKG (1:2000, ADI-KAP-PK005-F, Enzo Life Sciences), vasodilator-stimulated phosphoprotein (VASP) and phospho-VASP (1:1000, 3112 and 1:2000, 3114, Cell Signaling, Danvers, MA, USA) as marker of PKG activity, profibrotic mediator transforming growth factor (TGF)-β1 (1:250, SC-146, SantaCruz Biotechnology), MMP-2 (1:5000, NB200-193, Novus Biologicals) and MMP-9 (1:1000, SC-6840, SantaCruz Biotechnology). After washing, membranes were incubated in horseradish peroxidase-conjugated secondary antibody. Immunoblots were developed by enhanced chemiluminescence detection. Protein band densities were quantified using GeneTools software (Syngene, Frederick, MD, USA). After adjusting protein band densities to GAPDH (1:10000, MAB374, Millipore, Billerica, MA, USA), band density values were normalised to the average value of the Co group for statistical analysis. Detailed description of immunoblot analysis is available as Additional file 1.

### Histology, immunohistochemistry

LV sections were stained with hematoxylin-eosin (H&E) and Masson’s trichrome (MT) to examine histopathological characteristics and interstitial myocardial fibrotic remodelling, respectively. To evaluate cardiomyocyte hypertrophy, transverse transnuclear widths (cardiomyocyte diameter) were measured of 100 longitudinally oriented, mono-nucleated cardiomyocytes on H&E stained LV sections cut on the same plane. Immunohistochemistry for the fibrosis marker fibronectin (Sigma-Aldrich, Budapest, Hungary), for the profibrotic mediator TGF-β1 (Santa Cruz Biotechnology), for the sGC derived second messenger cGMP (AbD Serotec, Düsseldorf, Germany) and for the nitro-oxidative stress marker nitrotyrosine (NT) (Millipore) were performed and analysed. Histological and immunohistochemical analyses were performed by two blinded observers. For detailed description see Additional file 1.

### Terminal deoxynucleotidyl transferase dUTP nick end labeling (TUNEL) assay

TUNEL assay was performed to detect DNA strand breaks using a commercial kit (DeadEnd™ Colorimetric TUNEL System, Promega, Mannheim, Germany). TUNEL positive cell nuclei were counted in 20 randomly selected fields of each section by two blinded observers. Data were normalised to the mean value of the Co group for statistical analysis.

### Drugs

The sGC activator cinaciguat (BAY 58-2667; 4-({(4-carboxybutyl)[2-(2-{[4-(2-phenylethyl)benzyl]oxy}phenyl)ethyl]amino}methyl)benzoic acid) was provided by Bayer HealthCare (Wuppertal, Germany) for oral application. Cinaciguat was suspended in 0.5 % methylcellulose solution. STZ was purchased from Sigma-Aldrich (Taufkirchen, Germany).

### Statistics

Data are presented as means and standard errors of the mean (SEM). After testing normal distribution of the data, two-factorial analysis of variance (ANOVA) (with diabetes and drug treatment as factors) was carried out. A P value of <0.05 was used as a criterion of significance. Where F value of diabetes × treatment interaction reached the level of significance Tukey HSD post hoc testing was performed to evaluate differences between the groups. Data that did not show normal distribution were transformed logarithmically before performing two-factorial ANOVA.

## Results

### Body weight, heart weight (HW) and glucose levels

All animals survived the study period and reached the end-point of the investigation. HW and BW significantly decreased in both diabetic groups while HW to BW ratio increased in the diabetic groups (Table 1). When compared with controls, DM led to significantly increased blood glucose levels and daily water intake. Cinaciguat treatment in diabetic rats did not influence blood glucose levels, but led to attenuated water intake (Table 1). Time-course of body weight changes is available as Additional file 2: Figure S1.

**Table 1:** Basic characteristics of study groups

Characteristic	Co	CoCin	DiabCo	DiabCin	P_diabetes_	P_treatment_	P_interaction_
Blood glucose (mmol/l)	5.8 ± 0.1	6.2 ± 0.1	30.8 ± 0.5*	29.3 ± 1.4*	<0.001	0.460	0.209
Water intake (ml/gBW/day)	0.078 ± 0.002	0.090 ± 0.002	0.787 ± 0.006*	0.597 ± 0.013*^†^	<0.001	<0.001	<0.001
Heart weight (g)	1.20 ± 0.06	1.24 ± 0.07	0.91 ± 0.04*	0.84 ± 0.03*	<0.001	0.805	0.278
Body weight (g)	480.7 ± 17.6	477.8 ± 20.9	293.5 ± 11.1*	247.6 ± 14.8*	<0.001	0.150	0.202
Heart weight/body weight	0.249 ± 0.008	0.259 ± 0.008	0.311 ± 0.009*	0.348 ± 0.015*	<0.001	0.026	0.196

The values of blood glucose, daily water intake, heart weight, body weight (BW) and heart weight to body weight ratio are shown of the study groups—vehicle-treated controls (Co), cinaciguat-treated controls (CoCin), vehicle-treated diabetic (DiabCo) and cinaciguat-treated diabetic (DiabCin) groups. Values are mean ± SEM of 10–12 experiments per group

* P < 0.05 vs. Co

^†^P < 0.05 vs. DiabCo (Tukey HSD test)

### Effects of cinaciguat on plasma and myocardial cGMP levels in DM

**Fig. 1:**
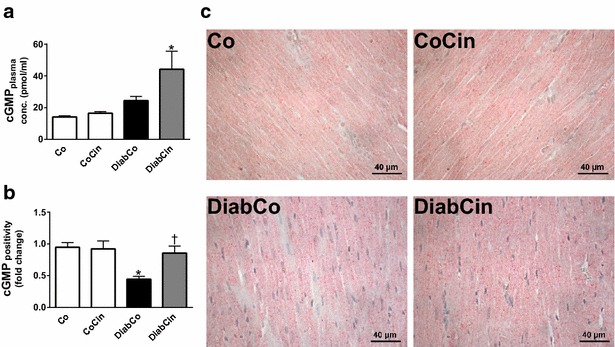
Effect of diabetes mellitus (DM) and cinaciguat treatment on plasma and myocardial cyclic guanosine monophosphate (cGMP) content. **a** Result of plasma cGMP enzyme immunoassay. **b** Quantification of cGMP (P value of diabetes × treatment interaction in the two-factorial ANOVA, P_interaction_ = 0.025) immunohistochemistry. **c** Representative images of cGMP immunohistochemistry. Magnification: ×400, *Scale bar* 40 µm. Groups: vehicle-treated control (Co), cinaciguat-treated control (CoCin), vehicle-treated diabetic (DiabCo) and cinaciguat-treated diabetic (DiabCin). Bar graphs represent mean ± SEM of 9–11 experiments per group. *P < 0.05 vs. Co, ^†^P < 0.05 vs. DiabCo (Tukey HSD test)

Cinaciguat treatment had no effect on plasma cGMP levels in control animals (Fig. 1a), however it resulted in a pronounced increase of plasma cGMP in DM (Fig. 1a). According to cGMP immunohistochemistry, the cGMP content of LV myocardium was significantly lower in DM than in controls (Fig. 1b, c). However, chronic treatment with cinaciguat restored cGMP to the control level (Fig. 1b, c).

### Effects of diabetes mellitus and cinaciguat treatment on myocardial nitro-oxidative stress

**Fig. 2:**
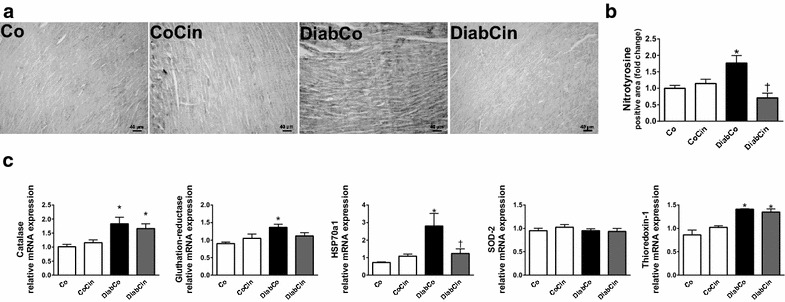
Cinaciguat treatment alleviates diabetes mellitus related oxidative stress. **a** Representative images of nitrotyrosine (NT) immunohistochemistry. Magnification: ×200, *Scale bar* 40 µm. **b** Quantification of NT immunohistochemistry (P value of diabetes × treatment interaction in the two-factorial ANOVA, P_interaction_ <0.001) (n = 9–11/group). (**c**) Relative mRNA expression of catalase, gluthatione-reductase (P_interaction_ = 0.046), heat shock 70 kD protein 1A (HSP70a1) (P_interaction_ = 0.021), superoxide dismutase (SOD)-2 and thioredoxin-1 (n = 5–6/group). Groups: vehicle-treated control (Co), cinaciguat-treated control (CoCin), vehicle-treated diabetic (DiabCo) and cinaciguat-treated diabetic (DiabCin). *Bar graphs* represent mean ± SEM. *P < 0.05 vs. Co, ^†^P < 0.05 vs. DiabCo (Tukey HSD test)

DM was associated with increased NT immunoreactivity in LV myocardium referring to pronounced nitro-oxidative stress which was significantly alleviated by cinaciguat treatment (Fig. 2a, b). Increased expression of HSP70a1, catalase, gluthatione-reductase and thioredoxin-1 was found in DM (Fig. 2c). HSP70a1 and gluthatione-reductase mRNA expression in the DiabCin group remained on the level of healthy controls (Fig. 2c). SOD-2 expression did not show any difference among the groups (Fig. 2c).

### Cinaciguat protects against DM related alteration of the NO-sGC-cGMP-PKG signalling

**Fig. 3:**
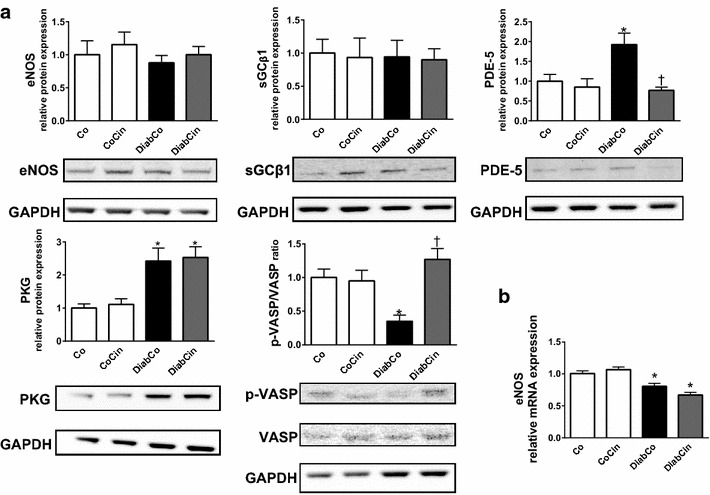
The effect of diabetes mellitus and cinaciguat on myocardial NO-sGC-cGMP-PKG signalling. **a** Relative protein expression and representative immunoblot bands of endothelial nitric oxide synthase (eNOS), soluble guanylate cyclase β1 (sGC β1), phosphodiesterase 5A (PDE-5) (P value of diabetes × treatment interaction in the two-factorial ANOVA, P_interaction_ = 0.024), protein kinase G (PKG), vasodilator-stimulated phosphoprotein (VASP) to phospho-VASP (p-VASP) ratio (P_interaction_ = 0.003). **b** Relative gene expression of eNOS (P_interaction_ = 0.039). Groups: vehicle-treated control (Co), cinaciguat-treated control (CoCin), vehicle-treated diabetic (DiabCo) and cinaciguat-treated diabetic (DiabCin).* Bar graphs* represent mean ± SEM of 5–6 experiments per group.*P < 0.05 vs. Co, ^†^P < 0.05 vs. DiabCo (Tukey HSD test)

Protein expression of eNOS and sGC β1 did not differ between healthy and diabetic rats (Fig. 3a) while eNOS gene expression was significantly lower in both diabetic groups (Fig. 3b). We detected elevated PDE-5 and PKG protein expression in the DiabCo group (Fig. 3a) whereas the p-VASP/VASP ratio (marker of PKG activity) was significantly reduced, showing severe deterioration of PKG signalling in DM (Fig. 3a). Application of cinaciguat in diabetic animals significantly reduced the expression of PDE-5, markedly increased PKG activity (as indicated by elevated p-VASP/VASP ratio) (Fig. 3a), while the expression of PKG did not differ between the two diabetic groups (Fig. 3a).

### Cinaciguat treatment protects against DM related fibrotic remodelling of the myocardium

**Fig. 4:**
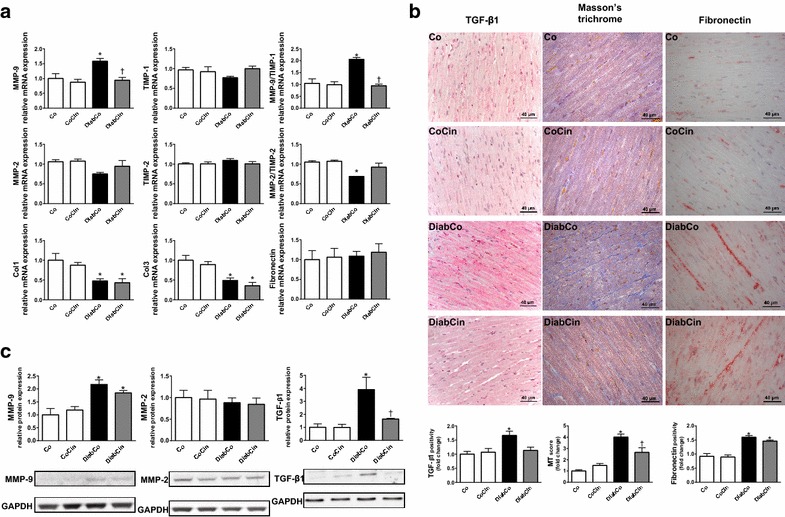
Effects of diabetes mellitus and cinaciguat on myocardial fibrotic remodelling. **a** Relative gene expression values of matrix metallopeptidase (MMP)-9 (P value of diabetes × treatment interaction in the two-factorial ANOVA, P_interaction_ = 0.038), tissue inhibitor of MMP (TIMP)-1, MMP-9/TIMP-1 ratio (P_interaction_ < 0.001), MMP-2, TIMP-2, MMP-2/TIMP-2 ratio, collagen 1a1 (Col1), 3a1 (Col3) and fibronectin (n = 5–6/group). **b** Representative images of Masson’s trichrome (MT) staining and representative immunohistochemical images of profibrotic mediator transforming growth factor (TGF)–β1 and fibrosis marker fibronectin. Quantification of MT staining (P_interaction_ = 0.002), TGF-β1 (P_interaction_ = 0.029) and fibronectin immunohistochemistry. Magnification: ×400,* Scale bar* 40 µm. (n = 9–11/group) **c** Relative protein expression and representative immunoblot bands of TGF-β1 (P_interaction_ = 0.049), MMP-2 and MMP-9. (n = 5–6/group) Groups: vehicle-treated control (Co), cinaciguat-treated control (CoCin), vehicle-treated diabetic (DiabCo) and cinaciguat-treated diabetic (DiabCin). *Bar graphs* represent mean ± SEM. *P < 0.05 vs. Co, ^†^P < 0.05 vs. DiabCo (Tukey HSD test)

DM was associated with dysregulation of the MMP system indicated by markedly increased MMP-9/TIMP-1 and reduced MMP-2/TIMP-2 gene expression ratios (Fig. 4a). These alterations were attenuated in the DiabCin group (Fig. 4a). Although fibronectin expression remained unchanged, Col1 and Col3 expression levels were significantly lower in both diabetic groups (Fig. 4a). The profibrotic TGF-β1 showed increased expression in the diabetic animals, which was significantly ameliorated by cinaciguat (Fig. 4c). Expression of MMP-9 showed a twofold increase in DM, while MMP-2 remained unchanged (Fig. 4c). Cinaciguat did not significantly alter these parameters (Fig. 4c). We found severe interstitial fibrosis of diabetic myocardium indicated by increased MT staining (Fig. 4c). Additionally, elevated immunoreactivity was observed against the profibrotic mediator TGF-β1 and fibrosis marker fibronectin in the diabetic heart (Fig. 4c). Application of cinaciguat reduced MT staining intensity of diabetic myocardium (Fig. 4c) while TGF-β1 immunoreactivity strongly tended to decrease (P = 0.051) (Fig. 4c).

### DM related myocardium hypertrophy and apoptosis is alleviated by cinaciguat

**Fig. 5:**
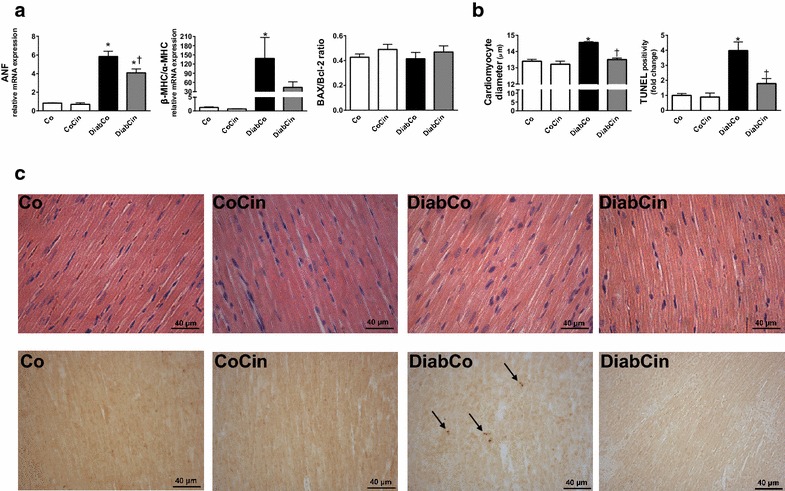
Effects of diabetes mellitus and cinaciguat on myocardial hypertrophy and apoptosis. **a** Relative mRNA expression of pathological hypertrophy markers atrial natriuretic factor (ANF) (P value of diabetes × treatment interaction in the two-factorial ANOVA, P_interaction_ = 0.036), β myosin heavy chain (MHC) to α-MHC ratio and the apoptosis marker Bcl2-associated X protein (BAX) to B-cell CLL/lymphoma 2 (Bcl-2) ratio (n = 5–6/group). **b** Mean cardiomyocyte diameter (P_interaction_ = 0.002) (as marker of cardiomyocyte hypertrophy) and quantification of TUNEL-positive cardiomyocyte nuclei (P_interaction_ = 0.009). (n = 9–11/group). **c** Representative images of hematoxylin—eosin stained sections and TUNEL assay of the left ventricle. Magnification: ×400,* Scale bar* 40 µm. Groups: vehicle-treated control (Co), cinaciguat-treated control (CoCin), vehicle-treated diabetic (DiabCo) and cinaciguat-treated diabetic (DiabCin).* Bar graphs* represent mean ± SEM. *P < 0.05 vs. Co, ^†^P < 0.05 vs. DiabCo (Tukey HSD test)

Myocardial mRNA expression data from LV myocardium of diabetic rats showed a marked increase of ANF and the β-MHC/α-MHC ratio (Fig. 5a). Treatment with cinaciguat caused a significant decrease of ANF expression in DM (Fig. 5a) while β-MHC/α-MHC ratio showed slight decrease (Fig. 5a). When compared with controls, increased cardiomyocyte width was observed in the DiabCo group indicative of cardiomyocyte hypertrophy. This increase was completely prevented by cinaciguat (Fig. 5b, c). The mRNA expression levels of proapoptotic BAX and antiapoptotic Bcl-2 did not differ among the study groups resulting in unchanged BAX/Bcl-2 ratio (Fig. 5a). DM was associated with increased TUNEL positivity in LV myocardium referring to pronounced DNA fragmentation (Fig. 5b, c). TUNEL positivity was effectively reduced in the DiabCin group (Fig. 5b, c).

### In vivo cardiac function is improved by cinaciguat in DM

In comparison with non-diabetic controls DiabCo group showed remarkably reduced MAP, LVSP, EF, SW, dP/dt_max_ and impaired dP/dt_min_ values, while LVEDP and Tau increased, indicating LV systolic and diastolic dysfunction (Table 2). HR significantly decreased in the diabetic groups while CO was not significantly different among the study groups (Table 2). MAP, LVSP, SW, dP/dt_max_ and dP/dt_min_ remained unchanged in the DiabCin group, however drug treatment markedly improved LVEDP and Tau in DM (Table 2). As a result of cinaciguat treatment EF tended towards improvement (P = 0.054) in DM (Table 2).

**Table 2:** Basic hemodynamic data of the study groups

Variable	Co	CoCin	DiabCo	DiabCin	P_diabetes_	P_treatment_	P_interaction_
HR (beats/min)	231 ± 11	246 ± 12	208 ± 8*	204 ± 10*	0.003	0.608	0.351
MAP (mmHg)	80.0 ± 2.0	81.0 ± 3.5	63.7 ± 2.5*	64.4 ± 3.1*	<0.001	0.845	0.969
LVSP (mmHg)	99.5 ± 2.6	103.5 ± 2.1	85.5 ± 1.3*	82.3 ± 2.3*	<0.001	0.858	0.102
SW (mmHg µl)	14,561 ± 1060	13,293 ± 948	9789 ± 592*	12,032 ± 1067	0.003	0.611	0.072
CO (µl/min)	42,347 ± 2472	41,306 ± 2804	33,360 ± 2162	38,605 ± 4288	0.057	0.484	0.297
EF (%)	70.42 ± 2.50	68.17 ± 2.70	58.09 ± 2.54*	68.02 ± 2.56	0.021	0.146	0.024
dP/dt_max_ (mmHg/s)	6539 ± 240	6804 ± 188	4933 ± 207*	4785 ± 230*	<0.001	0.791	0.350
dP/dt_min_ (mmHg/s)	−6135 ± 362	−6570 ± 446	−3883 ± 133*	−3723 ± 248*	<0.001	0.679	0.374
LVEDP (mmHg)	7.0 ± 0.6	7.2 ± 0.4	9.7 ± 0.7*	6.8 ± 0.3†	0.026	0.082	0.034
Tau (Weiss; ms)	10.3 ± 0.3	10.1 ± 0.3	17.3 ± 0.8*	14.9 ± 0.6*†	<0.001	0.016	0.054

Heart rate (HR), mean arterial pressure (MAP), maximal left ventricular (LV) systolic pressure (LVSP), stroke work (SW), cardiac output (CO), ejection fraction (EF), maximal slope of systolic pressure increment (dP/dt_max_) and diastolic pressure decrement (dP/dt_min_), LV end-diastolic pressure (LVEDP) and time constant of LV pressure decay (Tau) are shown. Groups: vehicle-treated controls (Co), cinaciguat-treated controls (CoCin), vehicle-treated diabetic (DiabCo) and cinaciguat-treated diabetic (DiabCin) animals. Values are mean ± SEM of 9–11 experiments per group

* P < 0.05 vs. Co

^†^P < 0.05 vs. DiabCo (Tukey HSD test)

**Fig. 6:**
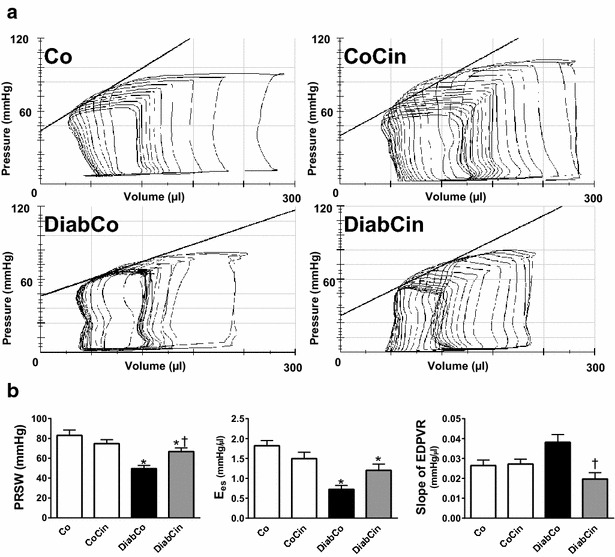
Effect of cinaciguat on left ventricular (LV) contractility and cardiac stiffness in diabetes mellitus. **a** Representative pressure–volume (P–V) loops, **b** preload recruitable stroke work (PRSW) (P value of diabetes × treatment interaction in the two-factorial ANOVA, P_interaction_ = 0.003), slope (E_es_) of LV end-systolic P–V relationship (P_interaction_ = 0.006) and the slope of LV end-diastolic P–V relationship (EDPVR) (P_interaction_ = 0.004) are presented in vehicle-treated control (Co), cinaciguat-treated control (CoCin), vehicle-treated diabetic (DiabCo) and cinaciguat-treated diabetic (DiabCin) groups.* Bar graphs* represent mean ± SEM of 9–11 experiments per group. *P < 0.05 vs. Co, ^†^P < 0.05 vs. DiabCo (Tukey HSD test)

The values of load-independent, P–V-loop derived contractility indexes (E_es_, PRSW) were significantly reduced in diabetic animals indicating severe contractile dysfunction (Fig. 6a, b) Treatment with cinaciguat led to a significant increase in PRSW (Fig. 6b) while E_es_ showed a strong tendency towards improvement in DM (P = 0.092) (Fig. 6b).

In comparison with Co group the slope of EDPVR tended to increase (P = 0.063) in diabetic animals, which was significantly reduced by cinaciguat (Fig. 6b). Cinaciguat had no hemodynamic effects in non-diabetic rats.

## Discussion

In the current study we investigated the effects of the sGC activator cinaciguat on DM related cardiac alterations in an experimental type-1 diabetic animal model. We found that chronic application of cinaciguat did not affect blood glucose levels, however it effectively raised cGMP levels and restored cGMP-PKG signalling in the myocardium. These molecular changes were associated with attenuated cardiomyocyte hypertrophy, less severe fibrotic remodelling and markedly improved systolic and diastolic cardiac performance.

Rat model of STZ induced type-1 DM is widely used in research studies aiming at the characterisation of diabetic cardiomyopathy and its possible new therapeutic approaches. STZ is taken up via glucose transporter 2 to the pancreatic beta cells which ensures its selective toxicity for beta cells and the ability to induce insulin-dependent DM. In the present study increased blood glucose and daily water intake levels indicated obvious evidence of pronounced DM in STZ-treated rats which was concordant with earlier data [31].

The most important cause in DM that presumably leads to severe complications, including diabetic cardiomyopathy, is hyperglycemia. The imbalance in glucose homeostasis triggers numerous mechanisms and signalling pathways which result in cardiovascular dysfunction. Despite the fact, that many studies investigated its pathological background so far, the exact pathomechanisms of diabetic cardiomyopathy are still poorly understood. It is known that hyperglycemia increases production of ROS and RNS and subsequent upregulation of endogenous antioxidant systems and overexpression of heat shock proteins [32] through several mechanisms [1]. As a marker of excessive nitro-oxidative stress [33] a significantly higher NT positivity was shown in the diabetic myocardium which might have contributed to the observed induction of endogenous antioxidant systems (see thiorexodin-1, catalase or gluthatione-reductase gene expression levels) and to a remarkable heat shock response (increased HSP70a1 level). Pronounced nitro-oxidative stress can lead to the oxidation of the sGC enzyme which causes the loss of its heme prosthetic group and results in a NO-insensitive (inactive) form of sGC. Additionally, an imbalance of the different types of phosphodiesterases was presented in the failing heart which might have contributed to the rapid degradation of intracellular cGMP [34]. Decreased cGMP levels and subsequent impairment of NO-cGMP-PKG signalling is a well-known feature occuring in the diabetic myocardium. This observation was confirmed by our results showing reduced myocardial cGMP content in diabetic rats while sGC β1 and eNOS protein expression remained unchanged. Moreover, NO-cGMP-PKG signalling showed a severe deterioration in the diabetic group indicated by a reduced gene expression level of eNOS, increased PDE-5 protein levels (in accordance with previous data [35]) and significantly reduced phosphorylation of the PKG target VASP (lower p-VASP/VASP ratio, an index of PKG activity [23]). However, elevated PKG protein expression was observed which might reflect an ineffective compensatory mechanism in the LV. The presumable loss of the widely described cGMP related cytoprotection was associated with a four times higher extent of DNA damage in DM compared with controls (indicated by the TUNEL reaction). The observed discrepancy between impairment of myocardial cGMP-signalling and unchanged plasma cGMP levels in DiabCo vs. Co animals might be the consequence of diabetes-associated increase in ANF levels and the subsequent activation of natriuretic peptide receptors and particulate guanylate cyclase (pGC) in organs other than the heart, as plasma cGMP is seen as an overspill of intracellular cGMP from various tissues [36].

The sGC activator cinaciguat has been described to exert disease specific effects since it preferentially activates the oxidised, inactive form of the enzyme (that is present mainly in diseased conditions) thus restores its cGMP producing ability [17] in various pathological conditions associated with excessive nitro-oxidative stress [19, 21]. With the application of cinaciguat we achieved massive cytoprotective effects in DM, that was confirmed by lower NT staining (indicative of attenuated nitro-oxidative stress), normalised expression of endogenous antioxidant and heat shock protein systems as well as lower extent of DNA breaks (less severe TUNEL positivity). The mechanism by which cinaciguat decreased nitro-oxidative stress remains unclear and needs further investigations. A possible explanation might be the cGMP-induced down-regulation of NADPH-oxidases, as proposed by a recent experimental work [37]. By reducing oxidative damage to the myocardium with cinaciguat, pathologically high levels of PDE-5 enzyme reversed to the level of control animals, which might have contributed to the accumulation of cGMP in the diabetic heart and plasma, too. The augmented cGMP production consequently reinforced the impaired PKG activity (proven by markedly increased myocardial p-VASP/VASP ratio) that further contributed to the cardioprotective effects of cinaciguat in DM.

Pathological remodelling is a well-known phenomenon in the diabetic myocardium, however there are several remaining questions regarding the underlying mechanisms to be clarified. A number of key pathophysiological features such as cardiac hypertrophy, fibrotic remodelling, myocardial tissue injury with DNA fragmentation are lying in the background of diabetic cardiomyopathy [1]. In our diabetic rat model pathological cardiomyocyte hypertrophy was developed demonstrated by increased cardiomyocyte diameter, increased ANF gene expression values and a shift in the β/α-MHC expression ratio. Chronic pharmacological activation of sGC showed antihypertrophic effects not only on the histological level (decreased cardiomyocyte diameter) but also in the molecular environment (significantly reduced ANF gene expression, strong tendency towards reversed shift in β/α-MHC ratio). Our results are concordant with data from a recent publication [23] demonstrating antihypertrophic and antifibrotic effects of cinaciguat in cultured cells. Fibrotic remodelling of the diabetic myocardium is strongly associated with dysregulation of MMPs [1], increased fibroblast proliferation [38], decreased Col1 and Col3 mRNA expression [39] and TGF-β1 signalling [40]. In accordance with previous literature data we found an upregulation of the profibrotic mediator TGF-β1, downregulation of Col1 and Col3 mRNA levels, intense MT and fibronectin staining (as fibrosis markers) and increased DNA fragmentation in diabetic cardiomyopathy. Data from our experiments revealed that treatment with the sGC activator cinaciguat effectively prevented pathological changes in the myocardium by improving MMP dysregulation (reverting altered MMP-9/TIMP-1 and MMP-2/TIMP-2 ratios), by decreasing myocardial level of TGF-β1 and preventing fibrotic changes, as well as repressing myocardial TUNEL positivity. Interestingly, we did not find any differences in BAX/Bcl-2 ratio among the study groups which suggest that classical apoptotic pathways are not involved in the observed beneficial effects. A possible explanation might be a cross-talk between sGC-cGMP axis and TGF-β-dependent extracellular signal-regulated kinase signalling (as reported by Beyer et al. [41]), however this hypothesis requires further investigations.

Several clinical and experimental studies have focused on the investigation of cardiac function in DM. Both systolic and diastolic dysfunctions have been described in type-1 diabetes in the literature using different non-invasive and invasive methods [2, 31]. We found similar alterations in our current study regarding DM related cardiac dysfunction. We observed a significant reduction of MAP (probably as a consequence of diabetic polyuria [42]) in DM compared with controls that was not affected by chronic cinaciguat treatment. Whether cinaciguat administration acutely affects MAP in diabetic rats, needs further non-invasive investigations. DiabCo group showed significantly deteriorated systolic function (as reflected by decreased LVSP, SW, EF and dP/dt_max_). EF and dP/dt_max_ are widely used to assess contractile function, however they are known to be affected by pre- and afterload [29]. Therefore, in the present study we determined P–V-loop derived, pre- and afterload independent contractility parameters (E_es_, PRSW) that are more informative on intrinsic myocardial contractile function [29]. The specific indexes of LV contractility showed severe systolic dysfunction in the DiabCo group. As a result of sGC activation a remarkable improvement of systolic function has been achieved in diabetic animals: a significant increase of PRSW clearly reflects improved LV contractility, whereas EF and SW were restored to the levels of healthy controls. When investigating diastolic function we found significantly deteriorated LV active relaxation (as shown by impaired dP/dt_min_ and prolonged Tau) and markedly elevated LV diastolic stiffness (indicated by higher LVEDP and strong tendency towards increased EDPVR) in DM compared with controls. Cinaciguat effectively improved not only active relaxation (by remarkably shortening Tau) but also diastolic stiffness (by reverting LVEDP and EDPVR values).

*Study limitations:* Results in the current study are limited to young, male, type-1 diabetic rats. Rats did not receive any glucose-lowering medication. Acute effects of pharmacological activation of sGC on hemodynamics were not investigated. A possible explanation of cinaciguat’s beneficial effects in DM could be the improvement of cardiac microvascular perfusion, however that needs further investigations.

## Conclusions

To our knowledge, this is the first study reporting the effects of pharmacological sGC activation on diabetic cardiomyopathy and DM related myocardial dysfunction. We confirmed that the sGC activator cinaciguat improves pathological features of diabetic cardiomyopathy without affecting blood glucose levels. Molecular mechanisms underlying the advantagous effects of sGC activation include the upregulation of NO-sGC-cGMP-PKG axis which might crosstalk with antioxidant, antihypertrophic and antifibrotic cascades hence preventing cardiac complications of diabetic metabolism and improving DM related cardiac dysfunction. Based on the data presented here pharmacological sGC activation might be a potential therapeutic approach to improve cardiovascular dysfunction in DM.

## Additional files

10.1186/s12933-015-0309-x Detailed description of material and methods used in this study.
10.1186/s12933-015-0309-x Time-course of body weight loss in diabetes mellitus. The graph shows the time-course of changes in body weight in vehicle-treated control (Co), cinaciguat-treated control (CoCin), vehicle-treated diabetic (DiabCo) and cinaciguat-treated diabetic (DiabCin) rats. Graph represent mean ± SEM, n = 9–11/group *P < 0.05 vs. Co (Tukey post hoc test).

## Abbreviations

α-MHC: myosin heavy chain alfa
ANF: atrial natriuretic factor
BAX: Bcl2-associated X protein
Bcl-2: B-cell CLL/lymphoma 2
β-MHC: myosin heavy chain beta
BW: body weight
Col1: Collagen 1a1
Col3: Collagen 3a1
CO: cardiac output
dP/dt_max_: maximal slope of systolic pressure increment
dP/dt_min_: maximal slope of diastolic pressure decrement
EDPVR: end-diastolic pressure–volume relationship
E_es_: slope of the left ventricular end-systolic pressure–volume relationship
EF: ejection fraction
EIA: enzyme immunoassay
eNOS: endothelial nitric oxide synthase
ESPVR: end systolic pressure–volume relationship
GADPH: glyceraldehyde-3-phosphate dehydrogenase
H&E: hematoxylin and eosin
HR: heart rate
HSP70a1: heat shock 70 kD protein 1A
HW: heart weight
LV: left ventricle
LVEDP: left ventricular end-diastolic pressure
LVSP: left ventricular systolic pressure
MAP: mean arterial pressure
MMP-2: matrix metallopeptidase 2
MMP-9: matrix metallopeptidase 9
MT: Masson’s trichrome
NO: nitric oxide
NT: nitrotyrosine
PDE-5: phosphodiesterase 5
PKG: protein kinase G
PRSW: preload recruitable stroke work
P–V: pressure–volume
ROS: reactive oxygen species
RNS: reactive nitrogen species
sGC: soluble guanylate cyclase
SOD-2: superoxide-dismutase 2
STZ: streptozotocin
SW: stroke work
TIMP-1: tissue inhibitor of matrix metallopeptidase 1
TIMP-2: tissue inhibitor of matrix metallopeptidase 2
VASP: vasodilator-stimulated phosphoprotein
