# Supplementary material for: The soluble guanylate cyclase activator cinaciguat prevents cardiac dysfunction in a rat model of type-1 diabetes mellitus
Source: Cardiovasc Diabetol. 2015 Oct 31;14:145. doi: 10.1186/s12933-015-0309-x (PMC4628236; doi:10.1186/s12933-015-0309-x)
Supplement: Supplementary file 1 — 10.1186/s12933-015-0309-x Detailed description of material and methods used in this study. [file 12933_2015_309_MOESM1_ESM.docx]

**SUPPLEMENTARY MATERIAL**

**The soluble guanylate cyclase activator cinaciguat prevents cardiac dysfunction in a rat model of type-1 diabetes mellitus**

**Methods**

*Hemodynamic measurements*

Invasive hemodynamic measurements were carried out as described previously [1]. Briefly, after the chronic treatment period, the animals were anesthetised with a mixture of ketamin (100 mg kg^-1^) and xylazine (3 mg kg^-1^) intraperitoneally, tracheotomised and intubated to facilitate breathing. Rats were placed on controlled heating pads, core temperature was maintained at 37 ºC. The left external jugular vein was cannulated with a polyethylene catheter for fluid administration. A 2F microtip pressure-conductance microcatheter (SPR-838, Millar Instruments, Houston, TX, USA) was inserted into the right carotid artery and advanced into the ascending aorta. After 5 min stabilisation, mean arterial blood pressure (MAP) and heart rate (HR) were recorded. Afterwards, the catheter was advanced into the left ventricle (LV) under pressure control. After 5 min stabilisation, signals were continuously recorded at a sampling rate of 1000 samples/s using a pressure-volume (P-V) conductance system (MPVS-Ultra, Millar Instruments) connected to the PowerLab 16/30 data acquisition system (AD Instruments, Colorado Springs, CO, USA), stored, and displayed on a personal computer by the LabChart5 Software System (AD Instruments). A special P-V analysis programme (PVAN, Millar Instruments) was used to compute and calculate mean arterial pressure (MAP), maximal LV systolic pressure (LVSP), LV end-diastolic pressure (LVEDP), maximal slope of systolic pressure increment (dP/dt_max_) and diastolic pressure decrement (dP/dt_min_), time constant of LV pressure decay (Tau; according to the Weiss method [2]), ejection fraction (EF), stroke work (SW) and cardiac output (CO). LV P-V relations were determined at different preloads during transient occlusion of the inferior caval vein. The slope (E_es_) of the LV end-systolic P-V relationships (ESPVR; according to the parabolic curvilinear model) and preload recruitable stroke work (PRSW) were calculated as load-independent indexes of LV contractility and the slope of the LV end-diastolic P-V relationship (EDPVR) was calculated as reliable index of LV diastolic stiffness.

After completing the hemodynamic measurements in vivo and in vitro volume calibrations were performed as described previously [2]. In vivo volume calibration was performed with hypertonic saline (100 µl) i.v. bolus injection. From the shift of P-V loops parallel conductance (Vp) was calculated and cardiac mass volume was corrected appropriately. Finally, in vitro cuvette calibration was carried out as follows: nine cylindrical holes in a block 1 cm deep and with known diameters (ranging from 2 to 11 mm) were filled with fresh heparinised whole blood of the animal. In this calibration, the linear volume-conductance regression of the absolute volume in each cylinder versus the raw signal acquired by the conductance catheter was used as the volume calibration formula [3].

*Myocardial mRNA analysis*

We performed quantitative real-time polymerase chain reaction (qRT-PCR) experiments as described previously [4]. Briefly, LV samples were harvested immediately after euthanasia, snap frozen in liquid nitrogen and stored at -80ºC. After homogenisation total RNA was isolated from LV tissue by using RNeasy Fibrous Tissue Kit (Qiagen, Hilden, Germany) according to the manufacturer’s instructions. RNA concentration was measured photometrically at 260 nm. RNA purity was ensured by obtaining a 260/280 nm optical density ratio of ∼2.0. Reverse transcription was performed with QuantiTect Reverse Transcription Kit (Qiagen) by using 1 µg RNA of each sample and random primers. All polymerase chain reactions (PCR) were performed on the StepOnePlus™ Real-Time PCR System (Applied Biosystems, Foster City, CA, USA). Every sample was quantified in triplicates in a volume of 10 µl in each well containing cDNA (1 µl), TaqMan^®^ Universal PCR MasterMix (5μl) and TaqMan^®^ Gene Expression Assay (Applied Biosystems) for the following targets (0.5 µl) (as indicated in Table 1.): atrial natriuretic factor (ANF), myosin heavy chain alfa (α-MHC) and beta (β-MHC)), collagen 1a1 (Col1), 3a1 (Col3) and fibronectin as pathological cardiac hypertrophy markers, antiapoptotic mediator B-cell CLL/lymphoma 2 (Bcl-2), proaptotic mediator Bcl2-associated X protein (BAX), endothelial nitric oxide synthase (eNOS), mediators of cardiac remodelling such as matrix metallopeptidase 2 (MMP-2) and MMP-9 and their endogenous inhibitors tissue inhibitor of matrix metallopeptidase (TIMP)-1 and TIMP-2, members of different antioxidant systems like catalase, thioredoxin-1, gluthatione-reductase, superoxide dismutase 2 (SOD-2) and heat shock 70kD protein 1A (HSP70a1) as a member of heat shock proteins. Gene expression data were normalised to glyceraldehyde-3-phosphate dehydrogenase (GAPDH) and expression levels were calculated using the CT comparative method (2^−ΔCT^). All results are expressed as values normalised to a positive calibrator (a pool of cDNAs from all samples of the Co group).

*Immunoblot analysis*

LV tissue samples were homogenized in radioimmunoprecipitation assay lysis buffer (RIPA; 50mM Tris HCl pH 8, 150 mM NaCl, 1% NP-40, 0.5% sodium deoxycholate, 0.1% SDS) containing Complete Protease Inhibitor Cocktail (Roche, Mannheim, Germany). Protein concentration was determined using the Pierce^®^ BCA Protein Assay Kit (Thermo Scientific, Rockford, IL, USA). Samples were mixed with 2× Laemmli buffer and boiled at 95ºC for 5 min. Equal amounts of protein (10–30 µg) were loaded and separated on commercial available precast 4–12% SDS-PAGE gel (NuPAGE^®^ Novex^®^ Bis-Tris Mini Gel, Invitrogen, Carlsbad, CA, USA). Afterwards, proteins were transferred to nitrocellulose membrane by semi-dry electroblotting system (iBlot™ Gel Transfer Device, Invitrogen). Membranes were blocked either in 5 % non-fat milk in Tris-buffered saline containing 0.1% Tween-20 (TTBS) or in 1 % bovine serum albumine in TTBS (MMP-9) for 1 h. After blocking, membranes were incubated overnight at 4ºC with primary antibodies (diluted in 1% bovine serum albumine in TTBS) against various targets proteins (as listed in Table 2.) as follows: members of NO signalling such as eNOS, soluble guanylate cyclase β1 (sGC β1), phosphodiesterase 5A (PDE-5), protein kinase G (PKG), vasodilator-stimulated phosphoprotein (VASP) and phospho-VASP as marker of PKG activity, the profibrotic mediator transforming growth factor-β1 (TGF-β1) and MMP-2 and MMP-9. After washing, membranes were incubated in horseradish peroxidase (HRP) – conjugated secondary antibody dilutions at room temperature (RT) for 1 h (anti-rabbit IgG, anti-mouse IgG, anti-goat IgG appropriately, 1:2000, Cell Signaling, Danvers, MA, USA). Immunoblots were developed using Pierce^®^ ECL Western Blotting Substrate Kit (Thermo Scientific). Protein band densities were quantified using GeneTools software (Syngene, Frederick, MD, USA). GAPDH was used to assess equal protein loading. Values of protein band densities (after adjusting to GAPDH band densities) were normalised to the average value of the Co group and were used to perform statistical analysis.

*Histology and immunohistochemistry*

Myocardial samples were collected for histological examination immediately after invasive hemodynamics, samples were fixed in 4% buffered paraformaldehyde (PFA) for 24 h, embedded in paraffin and 5 µm thick sections were cut. Hematoxylin and eosin (H&E) and Masson’s trichrome (MT) staining were carried out to examine histopathological characteristics and fibrotic remodelling of the LV in the study groups. Light microscopic examination was performed with a Zeiss microscope (Axio Observer.Z1, Carl Zeiss, Jena, Germany) and digital images were aquired using an imaging software (QCapture Pro 6.0, QImaging, Canada) at 400x magnification. To evaluate cardiomyocyte hypertrophy we measured the transverse transnuclear widths (cardiomyocyte diameter) of randomly selected 100 cardiomyocytes of the LV on H&E stained sections. To assess fibrotic remodelling semiquantitative scoring of MT sections was performed (Scores: 0-3; 0: no staining, 1: weak, 2: mild, 3: intense staining). Immunohistochemistry was performed as described previously [5]. Briefly, after antigen retrieval (citric acid buffer) slides were incubated with primary antibodies against fibrosis marker fibronectin (rabbit polyclonal anti-fibronectin, 1:1000, Sigma-Aldrich, Budapest, Hungary), the profibrotic mediator TGF-β1 (rabbit polyclonal anti-TGF-β1, 1:100; Santa Cruz Biotechnology, Santa Cruz, CA, USA) and the sGC derived second messenger cGMP (rabbit polyclonal anti-cGMP 1:2000, AbD Serotec, Düsseldorf, Germany). After that, slides were incubated with the secondary anti-rabbit antibodies (Biogenex SuperSensitive Link HK-9R, BioGenex, San Ramon, CA, USA) and developed using Fast Red (Dako, Glostrup, Denmark). Semiquantitative scoring was performed (area score 0: up to 10 % positive cells, 1: 11-50 % positive cells, 2: 50-80 % positive cells, 3: above 80 % positive cells; intensity score 1: weak, 2: mild, 3: strong, 4: very strong staining) by two blinded observers. In case of fibronectin area score, in case of TGF-β1 and cGMP area-intensity score (area score × intensity score) were calculated. Nitrotyrosine immunohistochemistry was performed in order to assess nitro-oxidative stress as described previously [6]. After deparaffinisation and antigen retrieval (0.1 mmol/L citrate buffer, pH 3, heating in microwave oven for 15 min) sections were incubated with polyclonal rabbit anti-nitrotyrosine antibody (1:200; overnight, 4 °C, Millipore, Billerica, MA, USA). HRP-conjugated avidin (Vectastain ABC kit, Vector Laboratories, Burlingame, CA, USA, 30 min, RT) and black colored nickel-cobalt enhanced diaminobenzidine (Vector Laboratories, 6 min, RT) were used to visualize the labeling. Images of five identical area of each section were taken using light microscope at 200x magnification (Zeiss AxioImager.A1 coupled with Zeiss AxioCAm MRc5 CCD camera, Carl Zeiss). Staining intensity was determined using ImageJ software (NIH, Bethesda, MD, USA). The percentage of positively stained tissue area to total area of each slide was calculated. Data were normalised to the average of Co group and used for statistical analysis. All histological/immunohistochemical images were analysed by two blinded observers.

*Terminal deoxynucleotidyl transferase dUTP nick end labeling (TUNEL) assay*

Paraffin embedded, 5 µm thick heart tissue sections were used to detect DNA strand breaks in LV myocardium. TUNEL assay was performed using a commercial available kit (DeadEnd™ Colorimetric TUNEL System, Promega, Mannheim, Germany) according to the manufacturer’s protocol. Sections were rehydrated, treated with 20 µg/ml Proteinase K to retrieve antigenic epitopes for antibody labeling (at RT, 10 min). After washing, sections were refixed in 4% PFA and treated with a mixture of 98 µl Equilibration Buffer, 1 µl Biotinylated Nucleotide Mix and 1 µl recombinant Terminal Deoxynucleotidyl Transferase (rTdT) enzyme (at 37ºC, 60 min) to incorporate biotinylated nucleotides at the 3’-OH DNA ends. After washing, 0.3% hydrogen peroxide (H_2_O_2_) was used to quench endogenous peroxidase activity.

HRP-labeled streptavidin and 3,3'-diaminobenzidine was used to detect incorporated nucleotides. TUNEL positive cell nuclei were counted by two blinded observers in 20 fields of each section at 400x magnification. Data were normalised to the mean value of the Co group and were used to perform statistical analysis.

**References**

[1] Radovits T, Bomicke T, Kokeny G, et al. The phosphodiesterase-5 inhibitor vardenafil improves cardiovascular dysfunction in experimental diabetes mellitus. Br J Pharmacol. 2009;156:909-919.

[2] Pacher P, Nagayama T, Mukhopadhyay P, Batkai S, Kass DA. Measurement of cardiac function using pressure-volume conductance catheter technique in mice and rats. Nat Protoc. 2008;3:1422-1434.

[3] Radovits T, Korkmaz S, Loganathan S, et al. Comparative investigation of the left ventricular pressure-volume relationship in rat models of type 1 and type 2 diabetes mellitus. Am J Physiol Heart Circ Physiol. 2009;297:H125-133.

[4] Radovits T, Olah A, Lux A, et al. Rat model of exercise-induced cardiac hypertrophy: hemodynamic characterization using left ventricular pressure-volume analysis. Am J Physiol Heart Circ Physiol. 2013;305:H124-134.

[5] Fang L, Radovits T, Szabo G, Mozes MM, Rosivall L, Kokeny G. Selective phosphodiesterase-5 (PDE-5) inhibitor vardenafil ameliorates renal damage in type 1 diabetic rats by restoring cyclic 3',5' guanosine monophosphate (cGMP) level in podocytes. Nephrol Dial Transplant. 2013;28:1751-1761.

[6] Masszi G, Benko R, Csibi N, et al. Endothelial relaxation mechanisms and nitrative stress are partly restored by Vitamin D3 therapy in a rat model of polycystic ovary syndrome. Life Sci. 2013;93:133-138.

**Table 1.** Identification numbers (ID) of TaqMan® Gene Expression Assays used in quantitative real time (qRT) PCR experiments

| Target gene | Abbreviation | Assay ID |
| --- | --- | --- |
| natriuretic peptide A | ANF | Rn00561661_m1 |
| myosin heavy chain alfa | α-MHC | Rn00568304_m1 |
| myosin heavy chain beta | β-MHC | Rn00568328_m1 |
| B-cell CLL/lymphoma 2 | Bcl-2 | Rn99999125_m1 |
| Bcl2-associated X protein | BAX | Rn02532082_g1 |
| catalase |  | Rn00560930_m1 |
| endothelial nitric oxide synthase | eNOS | Rn02132634_s1 |
| heat shock 70kD protein 1A | HSP70a1 | Rn04224718_u1 |
| matrix metallopeptidase 2 | MMP-2 | Rn01538170_m1 |
| matrix metallopeptidase 9 | MMP-9 | Rn00579162_m1 |
| superoxide dismutase 2 | SOD-2 | Rn00690587_g1 |
| thioredoxin-1 |  | Rn00587437_m1 |
| gluthatione-reductase |  | Rn01482159_m1 |
| tissue inhibitor of matrix metallopeptidase (TIMP) 1 | TIMP-1 | Rn00587558_m1 |
| TIMP metallopeptidase inhibitor 2 | TIMP-2 | Rn00573232_m1 |
| Collagen 1a1 | Col1 | Rn01463848_m1 |
| Collagen 3a1 | Col3 | Rn01437681_m1 |
| Fibronectin |  | Rn00569575_m1 |
| glyceraldehyde-3-phosphate dehydrogenase | GAPDH | Rn01775763_g1 |

The table shows the TaqMan® Gene Expression assays with the appropiate target mRNA transcripts used in qRT-PCR experiments.

**Table 2.** Primary antibodies used in Western blot procedures

| Target protein | Abbreviation | Primary antibody | Dilution | Molecular mass |
| --- | --- | --- | --- | --- |
| endothelial nitric oxide synthase | eNOS | SC-654 (SantaCruz Biotechnology, Santa Cruz, CA, USA) | 1:1000 | 140 kDa |
| soluble guanylate cyclase β1 | sGC β1 | NB100-91798 (Novus Biologicals, Cambridge, UK) | 1:1000 | 70 kDa |
| phosphodiesterase 5A | PDE-5 | ALX-210-099 (Enzo Life Sciences, Farmingdale, NY, USA) | 1:2000 | 130 kDa |
| protein kinase G | PKG | ADI-KAP-PK005-F (Enzo Life Sciences) | 1:2000 | 75 kDa |
| vasodilator-stimulated phosphoprotein | VASP | 3112 (Cell Signaling, Danvers, MA, USA) | 1:1000 | 50 kDa |
| phospho-VASP | p-VASP | 3114 (Cell Signaling) | 1:2000 | 50 kDa |
| matrix metallopeptidase 2 | MMP-2 | NB200-193 (Novus Biologicals) | 1:5000 | 62 kDa |
| matrix metallopeptidase 9 | MMP-9 | SC-6840 (SantaCruz Biotechnology) | 1:1000 | 92 kDa |
| transforming growth factor β1 | TGF-β1 | SC-146 (SantaCruz Biotechnology) | 1:250 | 25 kDa |
| glyceraldehyde-3-phosphate dehydrogenase | GAPDH | MAB374 (Millipore, Billerica, MA, USA) | 1:10000 | 38 kDa |

The table shows the primary antibodies (against various protein targets and reference protein glyceraldehyde-3-phosphate dehydrogenase) used in Western blot experiments.

**Figure legend:**

**Suppl. Fig. 1.** Time-course of body weight loss in DM

The graph shows the time-course of changes in body weight in diabetes mellitus.

Groups: vehicle-treated control (Co), cinaciguat-treated control (CoCin), vehicle-treated diabetic (DiabCo) and cinaciguat-treated diabetic (DiabCin). Graph represent mean±SEM, n=9-11/group *P<0.05 vs. Co (Tukey post hoc test)
