# Supplementary figures and images for: The soluble guanylate cyclase activator cinaciguat prevents cardiac dysfunction in a rat model of type-1 diabetes mellitus
Source: Cardiovasc Diabetol. 2015 Oct 31;14:145. doi: 10.1186/s12933-015-0309-x (PMC4628236; doi:10.1186/s12933-015-0309-x)

# Suppl. Fig. 1.

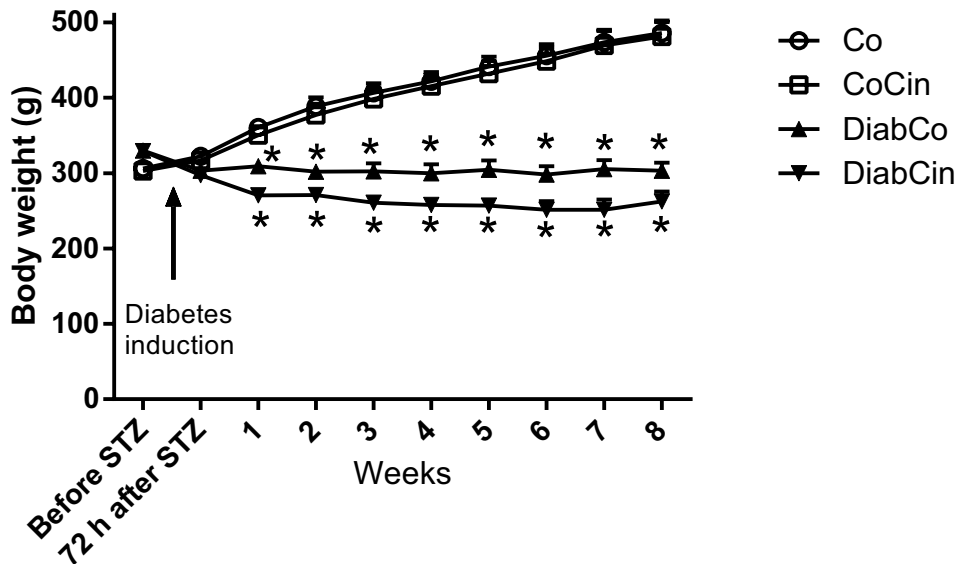

Supplement: Supplementary file 2 — 10.1186/s12933-015-0309-x Time-course of body weight loss in diabetes mellitus. The graph shows the time-course of changes in body weight in vehicle-treated control (Co), cinaciguat-treated control (CoCin), vehicle-treated diabetic (DiabCo) and cinaciguat-treated diabetic (DiabCin) rats. Graph represent mean ± SEM, n = 9–11/group *P < 0.05 vs. Co (Tukey post hoc test). [file 12933_2015_309_MOESM2_ESM.pdf]
